# Effect of incorporation of wheat bran, rice bran and banana peel powder on the mesostructure and physicochemical characteristics of biscuits

**DOI:** 10.3389/fnut.2022.1016717

**Published:** 2022-11-17

**Authors:** Wani Suhana Ayoub, Insha Zahoor, Aamir Hussain Dar, Nadira Anjum, R. Pandiselvam, Salma Farooq, Alexandru Vasile Rusu, João Miguel Rocha, Monica Trif, G. Jeevarathinam

**Affiliations:** ^1^Department of Food Technology, Islamic University of Science and Technology, Awantipora, India; ^2^Division of Food Science and Technology, Sher-e-Kashmir University of Agricultural Sciences and Technology, Jammu and Kashmir, India; ^3^Physiology, Biochemistry and Post-Harvest Technology Division, ICAR-Central Plantation Crops Research Institute (CPCRI), Kasaragod, India; ^4^Life Sciences Institute, University of Agricultural Sciences and Veterinary Medicine Cluj-Napoca, Cluj-Napoca, Romania; ^5^Faculty of Animal Science and Biotechnology, University of Agricultural Sciences and Veterinary Medicine Cluj-Napoca, Cluj-Napoca, Romania; ^6^Laboratory for Process Engineering, Environment, Biotechnology and Energy, Faculty of Engineering, University of Porto, Porto, Portugal; ^7^Associate Laboratory in Chemical Engineering, Faculty of Engineering, University of Porto, Porto, Portugal; ^8^Department of Food Research, Centre for Innovative Process Engineering (CENTIV) GmbH, Stuhr, Germany; ^9^Department of Food Technology, Hindusthan College of Engineering and Technology, Coimbatore, India

**Keywords:** biscuits, banana peel powder, wheat bran, rice bran, optical microscopy, storage

## Abstract

Various types of natural fiber-rich ingredients are added into bakery-based products to improve their fiber content for health promotional purposes. But the majority of these products usually include exotic dietary fiber components. The aim of this study was to develop biscuits incorporated with wheat bran, rice bran and banana peel powder and to evaluate the effects on physicochemical properties and sensory acceptability of these different biscuit samples. Wheat bran, rice bran and banana peel powder was used to substitute refined wheat flour in biscuit samples at different levels (0, 5, 10, 15, 20, 25, and 30%). The effect of wheat bran, rice bran and banana peel powder incorporation on proximate composition, physical characteristics, texture profile, color and sensory evaluation of biscuit samples were investigated. The moisture content of the product showed a significant (*p* ≤ 0.01) decreasing trend while as protein showed increasing trend with increasing level of incorporation of wheat bran, rice bran and banana peel powder. Also there was a considerable effect on L*(darkness to lightness), a*(greeness to redness), and b*(blueness to yellowness) values of biscuit samples. Among the physical parameters diameter and thickness decreased non-significantly (*p* ≤ 0.01) with the addition of different fibers whereas spread ratio and weight increases. Sensory attributes showed a significant (*p* ≤ 0.01) increasing trend with an increase in the level of incorporation of different fibers. Based on sensory evaluation biscuits prepared with 15% wheat bran, 15% rice bran, and 10% banana peel powder were rated best. The biscuits were packed in high density polyethylene (HDPE) boxes and were analyzed on different intervals *viz.* 0, 30, and 60th day. In samples of optimized biscuits, the ash content, protein, fat and color exhibited a non- significant tendency of declining over storage. It was discovered that the ash content dropped from0.86 to 0.67% in Wb^4^, 0.95 to 0.75% in Rb^4^, and 1.15to 0.92% in Bpp^3^. However there was a considerable increase in moisture content during storage.

## Introduction

Biscuits are readily available, bite-sized, inexpensive, and have a long shelf life, thus, they make a delicious adult snack. The fact that biscuits frequently include high quantities of fast digestible carbs, fats, and generally low levels of fiber precludes them from being viewed as a healthy snack despite of the fact that they are a very popular bakery item around the world (1, 2). Nowadays, numerous health products, including those that are sugar-free, low in calories, and high in fiber, are readily available. In order to address health issues, one of the current trends is to increase the amount of fiber in cereal products (3–5). To create high-fiber biscuits, several researchers have employed fiber sources like apple, lemon, and mango peel powder, apple, oat, rice, barley bran, and coconut (6–10).

Dietary fiber was recognized as a crucial element of a balanced diet in the 1980s, and the food industry started looking for tasty methods to boost the amount of fiber in their products (11). Each fraction of soluble or insoluble dietary fiber has different physiological impacts and nutritional benefits. Soluble fiber helps to decrease blood cholesterol and control blood sugar levels, whereas insoluble fiber encourages the passage of material through the digestive system (12). Cereal bran, which is high in insoluble fiber, and gums like pectin, which are rich in soluble fiber, have both been used in biscuits to increase the daily intake of fiber. Numerous researchers currently use cereal bran as a source of fiber to substitute refined flour in biscuits. One of the most plentiful and affordable sources of dietary fiber, vitamins, minerals, and bioactive substances is wheat bran, which is also regarded as a superior food component. Wheat bran consumption enhances health and guards against various diseases, such as colon cancer and cardiovascular conditions (13). Over time, the usage of wheat bran for human consumption has progressively risen (14) with increased focus in baked foods like biscuits. One of the nutrient-rich agricultural byproducts is rice bran. It is a mixture of lipids, proteins, fibers, minerals, and trace elements including calcium, magnesium, potassium, phosphorus, and manganese (15). The desire for new healthy meals is rising, including low-calorie, nutrient-rich, and high-fiber goods like biscuits. Rice bran, an essential source of dietary fiber, has been the subject of numerous studies to develop gluten-free products that can help to prevent a number of health conditions (16, 17). A growing body of research supports the idea that increasing dietary fiber consumption can help prevent illnesses including colon cancer, diverticulosis, diverticulitis, diabetes, and disorders of the gastrointestinal and cardiovascular system. Due to the medicinal potential of dietary fiber, new food items with fiber inclusion are being created. The creation of high-demand functional meals or foods with additional value will be facilitated by the addition of dietary fiber to a variety of products.

Fruit fibers including apple fiber, banana fiber, and dietary fiber from mango have been used in several researches (18). A tropical fruit, banana is grown in more than 122 nations worldwide. Banana peel waste is frequently disposed of in municipal landfills, aggravating already-existing environmental problems. However, by exploiting its high-value constituents, such as the dietary fiber fraction, which has a lot of potential in the creation of functional meals, this issue may be resolved. Utilizing banana peels by molding them into nutrient-rich foods like biscuits is one effort. According to findings from earlier studies, banana peels are a beneficial dietary item with a high fiber content. Banana peel flour may provide new goods with standardized ingredients for a variety of commercial uses (19). Wheat bran, rice bran and banana peel powder was found to be high in protein, dietary fiber, total phenolic content, antioxidant activity, and functional properties. These findings indicate that the powder made from these ingredients, along with flour, has a great deal of potential for use in the creation of new food formulations. Based on the foregoing studies, investigations on the physical characteristics, chemical composition, and organoleptic evaluation of the biscuits prepared with powdered banana peel have been conducted.

## Materials and methods

### Raw material

Bananas, refined wheat flour, sugar, butter, baking powder, milk powder, glucose, and vanilla were purchased from local market of Awantipora, India.

### Preparation of banana peel powder

**FIGURE 1:**
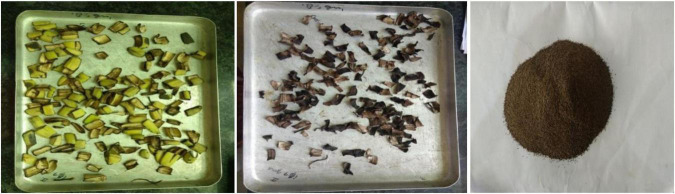
Preparation of banana peel powder.

The peels obtained from bananas (*Musa acuminate*) variety were removed and washed well by water. The peels were dried in hot air drier at 60°C for 24 h ground to soft powder, passed through a 0.60 mm mesh size and moisture content (5.83%). The banana peel powder was then stored in glass jar until use (20) (Figure 1).

### Preparation of biscuits

Banana peel powder, sugar powder, butter, skim milk powder, glucose, baking powder, vanilla flavor, and water were added to refined wheat flour to make biscuits (as required for proper consistency). First, creaming sugar, fat, and flavor (vanilla) then addition of refined wheat flour with (wheat bran, rice bran, and powdered banana peel) separately, skim milk powder, baking powder, and water containing glucose were all thoroughly combined with these ingredients. To prepare the dough, these components are combined for 2 min. The dough was sheeted by rolling it with a wooden rolling pin. In Morphy Richards’ (OTG 40 RC-55) oven, round biscuits were cut and cooked for 9–10 min at 180°C. Formulations used for preparation of biscuit samples are shown in Table 1.

**TABLE 1:** Formulations used for preparation of biscuit samples.

Ingredients	Different substitution levels of fibre (%)						
Refined wheat flour (g)	64	60.8	56.7	54.4	51.2	48	44.8
Wheat bran/rice bran/banana peel powder	0	5% (3.2 g)	10% (6.4 g)	15% (9.6 g)	20% (12.8 g)	25% (16 g)	30% (19.2%)
Sugar (g)	18	18	18	18	18	18	18
Shortening (g)	16	16	16	16	16	16	16
Milk powder (g)	1	1	1	1	1	1	1
Glucose (g)	1	1	1	1	1	1	1
Baking powder (g)	0.3	0.3	0.3	0.3	0.3	0.3	0.3
Vanillin extract (ml)	0.02	0.02	0.02	0.02	0.02	0.02	0.02

### Proximate analysis

Proximate parameters (moisture, fat, protein, fiber, and ash) were determined using the Association of Official Analytical Chemists (21) method. The nitrogen content of the samples was determined by micro-Kjeldhal method. The weight difference methods were used to determine moisture and ash content levels while crude fat of (refined wheat flour, wheat bran, rice bran, and banana peel powder) was determined by soxhlet method.

### Physical evaluation of biscuit samples

Physical characteristics were evaluated using the procedure of Smarzyński et al. (22). Using an analytical weighing scale, the weight of the biscuits was calculated as the average of the weights of six different biscuits. The weight’s average value was given in g. Using a digital vernier caliper, the diameter of four biscuit samples put edge to edge was measured to establish the thickness of the biscuits. Spread ratio was calculated by dividing diameter by thickness according to Bala et al. (23).

### Texture analysis of biscuits

With the following operating conditions, a texture analyzer (TA.HD.Plus, stable Micro systems, Godalming, Surrey, UK) was used to analyze the texture profile of biscuits. About 5 mm/s as the pre-test speed, 3 mm/s as the test speed, 10 mm/s as the post-test speed, 5 mm as the distance, and 2 cm as the distance between the supports (24). For each batch of biscuits, at least 10 measurements were obtained.

### Color measurement

The Hunter Lab Color flex (EZ Model No. 45/0) was filled with biscuit samples. The L*, a*, and b* were noted. L* designates lightness, whereas a* and b* stand for the red/green and yellow/blue values, respectively. Each sample underwent three measurements (25).

### Sensory evaluation

A 5-point hedonic scale approach was used to conduct sensory evaluation based on sensory qualities, with the following values: (5) excellent, (4) good, (3) average, (2) fair, and (1) poor. The semi-trained panel of five people from the department of food technology in Awantipora, Kashmir, evaluated the sensory quality of several biscuit samples based on color, flavor, texture, taste, and overall acceptability. The participants received the samples in a random sequence, along with distilled water to rinse their mouths after each sample tasting. The acquired scores of the examined qualities were used to determine overall acceptability, and four samples were selected for additional storage (26).

### Optical microscopy

The mesostructure of the samples was assessed using optical microscopy. The samples were deposited on a glass slide and observed under a stereomicroscope (SMZ 2B-2T, Nikon Corp., Japan). Images were then acquired using a digital camera (ToupCam™, Touptek Photonics, China).

### Statistical analysis

The data were averaged for three replications of each observation. The SPSS software (Version 17.0 for Windows, SPSS Inc., Chicago, USA) was used to do an analysis of variance with a significance threshold of 1% and use Duncan’s test to identify variations between means (27).

## Results and discussion

### Proximate composition of raw material

The samples were analyzed for chemical composition. The mean values of refined wheat flour, wheat bran, rice bran, and banana peel powder are presented in Table 2.

**TABLE 2:** Proximate composition of raw material.

Parameters	Refined wheat flour	Wheat bran	Rice bran	Banana peel powder
Moisture content (%)	12.5 ± 0.7^b^	2.43 ± 0.05^a^	9.2 ± 0.3^d^	5.83 ± 0.05^c^
Ash content (%)	0.36 ± 0.05^a^	4.5 ± 0.17^b^	9.86 ± 0.23^d^	9.66 ± 0.57^d^
Fat content (%)	0.73 ± 0.11^a^	5.4 ± 0.2^b^	12.33 ± 0.30^d^	5.8 ± 0.2^b^
Fiber content (%)	0.45 ± 0.09^a^	9.8 ± 0.2^b^	13 ± 0.2^c^	20.2 ± 0.5^d^
Protein content (%)	9.86 ± 0.15^b^	15.5 ± 0.15^a^	12.66 ± 0.15^d^	8.30 ± 0.17^a^

Data presented are Mean ± SD. Different superscript letters represent the *P* ≤ 0.05.

Table 2 shows the mean value of several proximate characteristics for refined wheat flour, wheat bran, rice bran, and banana peel powder. The results are in consistence with the findings of Ahluwalia et al. (28), Ayub et al. (29), and Siriamornpun et al. (30).

### Optimization of best formulation of biscuit samples

Any modified food’s success hinges on how well it passes sensory and how closely it resembles the original item. Most customers are unwilling to alter their preferences. Sensory analysis was used to found the best samples. To choose the top three formulations for additional research, all the samples were also submitted to physical characterization and textural examination.

### Evaluation of biscuit samples for selection of best formulation

Nineteen different formulations of biscuit samples were prepared from 3 different fiber incorporations. The samples obtained were analyzed for their sensory attributes, physical parameters, textural characteristics, and gross chemical composition as well as color.

### Sensory characteristics of biscuit samples

The sensory scores of biscuit samples with different levels of wheat bran, rice bran, banana peel powder are presented in Table 3. The sensory characteristics of all 19 biscuit samples (Control, Wb_2_, Wb_3_, Wb_4_, Wb_5_, Wb_6_, Wb_7_, Rb_2_, Rb_3_, Rb_4_, Rb_5_, Rb_6_, Rb_7_, Bpp_2_, Bpp_3_, Bpp_4_, Bpp_5_, Bpp_6_, Bpp_7_) were compared with control sample on the basis of color, flavor, texture, taste, and overall acceptability to select optimized product.

**TABLE 3:** Effect of wheat bran (Wb)/rice bran (Rb) and banana peel powder (Bpp) on the sensory quality of biscuit samples.

Formulations	Color	Flavor	Taste	Texture	Overall acceptability
Control (0%)	3.0 ± 0.00^de^	2.6 ± 0.48^cde^	3 ± 0.00^cdef^	2.5 ± 0.40^abcde^	2.9 ± 0.37^cdefg^
Wb_2_ (5%)	2.6 ± 0.43^cde^	2.65 ± 0.48^cde^	2.8 ± 0.40^cdef^	2.6 ± 0.75^bcde^	2.5 ± 0.43^cdef^
Wb_3_ (10%)	2.8 ± 0.39^cde^	2.7 ± 0.32^cde^	3.2 ± 0.50^cdef^	3.2 ± 0.26^def^	2.7 ± 0.38^cdefg^
Wb_4_ (15%)	3.0 ± 0.81^de^	3.2 ± 0.17^defg^	3.6 ± 0.48^ef^	3.4 ± 0.41^efg^	3.3 ± 0.41^efgh^
Wb_5_ (20%)	2.6 ± 0.48^cde^	2.8 ± 0.94^cdef^	2.5 ± 0.41^bcde^	3.0 ± 0.81^cdef^	2.6 ± 0.55^cdef^
Wb_6_ (25%)	2.2 ± 0.50^abcde^	2.9 ± 0.52^cdefg^	2.0 ± 00^abc^	3.2 ± 0.50^defg^	2.4 ± 0.44^bcdf^
Wb_7_ (30%)	2.2 ± 0.05^abcd^	2.2 ± 0.50^bcd^	2.2 ± 0.50^abcd^	2.4 ± 0.41^abcde^	2.3 ± 0.27^bcd^
Rb_2_ (5%)	3.6 ± 0.47^ef^	3.8 ± 0.25^fg^	3.3 ± 0.75^def^	3.5 ± 0.57^efg^	3.6 ± 0.33^gh^
Rb_3_ (10%)	3.6 ± 0.94^ef^	3.0 ± 0.81^cdefg^	3.0 ± 0.81^cdef^	3.6 ± 0.47^fg^	3.2 ± 0.25^defg^
Rb_4_ (15%)	4.5 ± 0.57^f^	4.0 ± 0.00^g^	3.8 ± 0.25^f^	4.1 ± 0.25^g^	4.2 ± 0.23^h^
Rb_5_ (20%)	2.7 ± 0.50^cde^	3.1 ± 0.25^defg^	2.2 ± 0.95^abcd^	2.7 ± 0.50^bcdef^	2.6 ± 0.58^cdef^
Rb_6_ (25%)	2.3 ± 0.75^bcde^	2.5 ± 0.57^cde^	2.5 ± 1.29^bcdf^	2.3 ± 0.17^abcd^	2.4 ± 0.49^bcd^
Rb_7_ (30%)	3.0 ± 1.41^de^	2.0 ± 0.81^bc^	2.0 ± 0.81^abc^	2.2 ± 0.50^abcd^	2.3 ± 0.73^bc^
Bpp_2_ (5%)	3.0 ± 0.00^de^	3.3 ± 0.47^efg^	3.0 ± 0.00^cdef^	3.0 ± 0.00^cdef^	2.9 ± 0.11^cdefg^
Bpp_3_ (10%)	3.1 ± 0.25^de^	3.0 ± 0.00^cdefg^	3.3 ± 0.25^def^	3.5 ± 0.57^efg^	3.4 ± 0.24^fgh^
Bpp_4_ (15%)	2.2 ± 0.50^abcde^	2.5 ± 0.40^cde^	2.0 ± 0.81^abc^	2.6 ± 0.75^bcdef^	2.3 ± 0.53^bcd^
Bpp_5_ (20%)	1.5 ± 0.57^abc^	1.5 ± 0.57^ab^	1.5 ± 0.57^ab^	2.0 ± 0.00^abc^	1.6 ± 0.58^ab^
Bpp_6_ (25%)	1.2 ± 0.50^ab^	1.0 ± 0.00^a^	1.0 ± 0.00^a^	1.7 ± 0.50^ab^	1.4 ± 0.16^a^
Bpp_7_ (30%)	1.0 ± 0.00^a^	1.0 ± 0.00^a^	1.2 ± 0.5^ab^	1.5 ± 0.57^a^	1.2 ± 0.19^a^

### Effect of wheat bran/rice bran/banana peel powder on color

**FIGURE 2:**
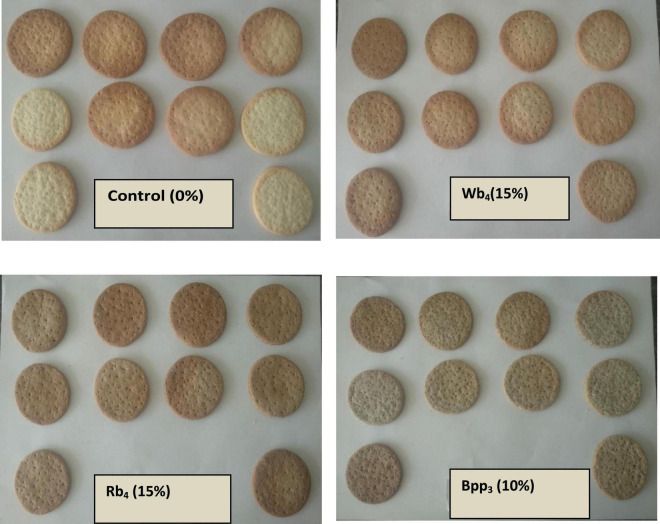
Control biscuits (0%) bran and optimized biscuit samples Wb_4_ (15%) wheat bran, Rb_4_ (15%) rice bran and Bpp_3_ (10%) banana peel powder at 60th day of storage.

In all of the treatments, the sensory score for biscuit color greatly improved as shown in Table 3. Wb4 (3), Rb4 (4.5), and Bpp3 (3.1) scored the highest mean scores, while Wb7 (2.2), Rb7 (2.37), and Bpp7 recorded the lowest (1). One important factor that significantly influences the quality of baked goods is the color property. A considerable change in color from light brown to darker shades of brown with more fiber substitution may be the result of non-enzymatic browning processes (Maillard reactions). Melanoidins, high-molecular-weight macromolecule substances, were created during baking as a result of this non-enzymatic process. Accordingly, the color of biscuits changes as the temperature and baking time increase and is influenced by the amount of sugars and proteins in the baking ingredients (31) (Figure 2).

### Effect of wheat bran/rice bran/banana peel powder on flavor

The score for flavor varied significantly with mean score of fiber biscuits (2.6) for control, (2.65) for Wb_2_, (2.7) for Wb_3_, (3.2) for Wb_4_, (2.8) for Wb_5_, (2.9) for Wb_6_, (2.2) for Wb_7_, (3.87) for Rb_2_, (3) for Rb_3_, (4) for Rb_4_, (3.1) for Rb_5_, (2.5) for Rb_6_, (2) for Rb_7_, (3.37) for Bpp_2_, (3) for Bpp_3_, (2.5) for Bpp_4_, (1.5) for Bpp_5_, (1) for Bpp_6_, and (1) for Bpp_7_ in Table 3. The maximum score for flavor was observed in these three samples Wb_4_, Rb_4_, and Bpp_3_. The flavor score for the control sample was found 2.6 and showed an increased trend in the other biscuit samples. However the flavor score gets reduced to (2.2, 2, 1) in samples like (Wb_7_, Rb_7_, Bpp_7_). Similar findings were also reported by Nagarajaiah et al. (32) and Jauharah et al. (33). The flavor and smell of the products depends on the volatile constituents of raw material (34).

### Effect of wheat bran/rice bran/banana peel powder on taste

Taste is the most desirable sensory attribute in bakery and confectionary group of food products. Therefore, average score of samples are presented in the Table 3. The score for taste varied non-significantly. Highest mean score value for taste was found in Wb_4_ (3.6), Rb_4_ (3.8), Bpp_3_ (3.3) and lowest in Wb_7_ (2.2), Rb_7_ (2), Bpp_7_ (1). The average mean score for taste got reduced in Wb_7_, Rb_7_, and Bpp_7_. The reason for the decrease may be the presence of tannins in banana peel powder, which causes bitter taste in biscuit samples, which further increased with increasing the concentration of wheat bran, rice bran, and banana peel powder. Tannin is present in banana peel powder and increases bitterness at the threshold level of poor after taste. This indicates that a higher level of incorporation of wheat bran, rice bran and banana peel powder in biscuits has influenced the taste adversely. Sudha et al. (6) reported decreased in the taste acceptability of cookies with increasing levels of rice or wheat bran. According to Bagheri and Seyedein (35), adding more rice or wheat bran to cookies made them less palatable. The biscuits which are produced with added wheat bran exhibit better sensory attributes, respectively they exhibit a smoother surface with an attractive color, specific aroma and taste which increases with the increase of wheat bran (36).

### Effect of wheat bran/rice bran/banana peel powder on texture

With increasing inclusion level throughout treatments, the texture of biscuit samples dramatically improved. Wb4 (3.4), Rb4 (4.12), and Bpp3 (3.5) had the highest texture scores, while Wb7 (2.4), Rb7 (2.2), and Bpp7 (1.5) received the lowest. The exceptionally high fiber content of wheat bran, rice bran, and powdered banana peel may be the cause of the product’s roughness. Texture of the biscuits depends mainly upon the rate of development of the dough and the proportion of sugar used (34).

### Effect of wheat bran/rice bran/banana peel powder on overall acceptability

**FIGURE 3:**
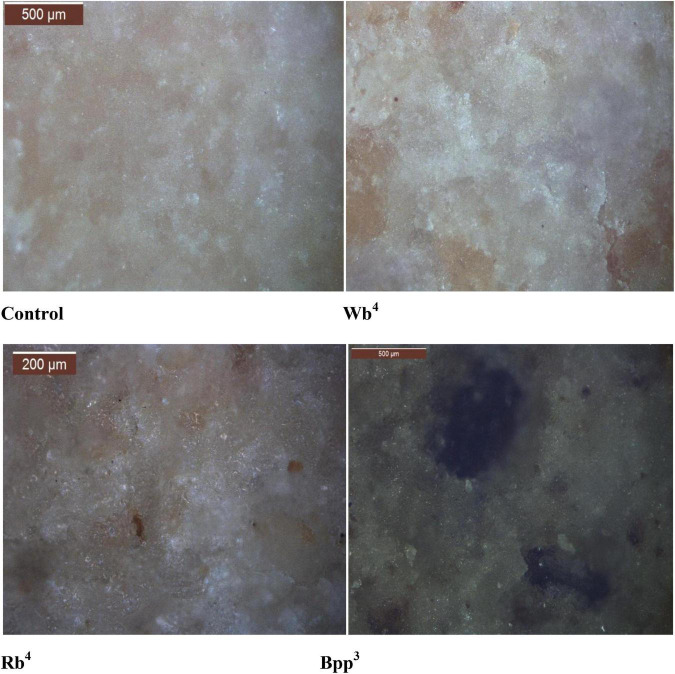
Optical Micrographs images of biscuit samples: Control, (Wb^4^), (Rb^4^), and Bpp^3^. Changes during storage (for 2 months) of optimized biscuit sample.

Table 3 lists the average ratings for the biscuits’ general acceptance. In comparison to the Control sample, Wb4, Rb4, and Bpp3 received the highest average ratings for color, flavor, taste, texture, and overall acceptability (Figure 3). Biscuits with up to 15% of refined wheat flour, wheat bran, or rice bran had satisfactory acceptance, as did biscuits with up to 10% of refined wheat flour, banana peel powder. These findings are supported by the findings of Murlidhar et al. (37).

### Physical characteristics of biscuit samples

Physical characteristics are crucial when choosing a packing material and creating a box. Physical characteristics of biscuits, in addition to their sensory attributes, aid in determining their acceptance by consumers. As a result, while the diameter, thickness, and weight were being measured, the spread ratio was determined using a formula.

### Effect of wheat bran/rice bran/banana peel powder on diameter

The results of the physical features of the biscuits were made using various ratios of chosen fibers displayed in Table 4. The average diameter (mm) measurements for all samples with various amounts of fiber inclusion revealed that the diameter of the biscuit samples did not change significantly over time. While the (Wb7, Rb7, and Bpp7) samples exhibited a decrease in diameter, the control sample showed an increase in diameter (50.5, 49.2, and 50.25 mm). Coleman et al. (38) as the amount of fiber integration increased, the diameter showed a decreasing trend because incorporation of dietary fiber improves the binding property of biscuit samples and prevents them to spread during baking (39).

**TABLE 4:** Effect of wheat bran/rice bran and banana peel powder on physical characteristics of biscuit samples.

Formulations	Diameter	Thickness	Weight	Spread ratio
Control	52 ± 0.5^f^	4.9 ± 0.50^d^	4.85 ± 0.05^a^	10.6
Wb_2_	50.7 ± 0.0^ef^	3.66 ± 0.81^cd^	4.97 ± 0.22^a^	14.5
Wb_3_	51.7 ± 0.5^def^	3.61 ± 0.50^bcd^	5.0 ± 0.20^a^	15.2
Wb_4_	51.5 ± 1.0^cdef^	3.55 ± 0.50^abcd^	5.02 ± 0.18^a^	15.4
Wb_5_	51.0 ± 1.1^bcdef^	3.5 ± 0.0^abcd^	5.27 ± 0.20^ab^	15.8
Wb_6_	50.7 ± 1.5^abcdef^	3.44 ± 0.50^abc^	5.32 ± 0.25^ab^	16.2
Wb_7_	50.5 ± 0.57^abcde^	3.38 ± 0.50^a^	5.55 ± 0.17^abc^	16.5
Rb_2_	50.5 ± 0.57^abcde^	4.7 ± 0.57^g^	4.90 ± 0.62^a^	10.85
Rb_3_	50.2 ± 0.5^abcd^	4.6 ± 0.57^efg^	5.86 ± 0.81^bcd^	10.97
Rb_4_	50 ± 0.0^abc^	4.55 ± 0.50^efg^	5.89 ± 0.26^bcd^	11.22
Rb_5_	49.7 ± 0.5^ab^	4.5 ± 0.0^ef^	6.05 ± 0.27^cd^	11.28
Rb_6_	49.5 ± 0.57^ab^	4.43 ± 0.57^e^	6.09 ± 0.16^cd^	11.34
Rb_7_	49.2 ± 0.5^a^	4.38 ± 0.50^e^	6.30 ± 0.52^d^	11.4
Bpp_2_	52 ± 0.81^ef^	3.66 ± 0.8^cd^	5 ± 0.21^a^	13.9
Bpp_3_	51.75 ± 0.5^def^	3.6 ± 1.0^abcd^	5.1 ± 0.18^a^	14.01
Bpp_4_	51.5 ± 0.57^cdef^	3.55 ± 1.2^abcd^	5.25 ± 0.25^ab^	14.29
Bpp_5_	50.7 ± 0.95^abcdef^	3.5 ± 0.81^abcd^	5.27 ± 0.51^ab^	14.5
Bpp_6_	50.5 ± 0.57^abcde^	3.43 ± 0.57^ab^	5.32 ± 0.25^ab^	14.7
Bpp_7_	50.25 ± 0.5^abcd^	3.38 ± 0.50^a^	5.55 ± 0.17^abc^	15.4

All values are average of three determinations (*n* = 3). Data presented are Mean ± SD. Value with same lower case letter in column are not significantly different (*p* ≤ 0.01).

### Effect of wheat bran/rice bran/banana peel powder on thickness

The control sample showed highest thickness of 4.9 while as the minimum thickness was measured in Wb_7_ (3.38), Rb_7_ (4.38), and Bpp_7_ (3.38). The thickness of biscuits decreased non-significantly with increased addition of fibers. The greater water-holding capacity of fibers may be the reason of this. According to Kohajdová et al. (40), adding larger amounts of apple fiber at 10% (5.51 mm) and 15% (5.33 mm) caused a meaningful reduction in the thickness of the cookies. Similar results were also confirmed by Shazia et al. (41). These results are also in close agreement with the findings of Singh et al. (42) who studied the effect of incorporating sweet potato flour with wheat flour on quality characteristics of cookies. They observed that thickness of cookies decreased from 48 to 40 mm with increase in sweet potato flour percentage.

### Effect of wheat bran/rice bran/banana peel powder on spread ratio

The biscuit spread ratio is a crucial quality indicator; the bigger the spread ratio, the heavier the product will be. Table 4 provides the average spread ratio values for the biscuit samples. As the quantity of fibers increases, it was observed that the spread ratio for different types of biscuit sample also increases. According to Murlidhar et al. (37), the difference between the control biscuit’s minimum spread ratio of 10.6 and the maximum spread ratios of Wb7, Rb7, and Bpp7’s (16.5, 11.4, and 15.4) could be explained by the control biscuit’s lower gas retention or by variations in the swelling patterns and rheological properties (43).

### Effect of wheat bran/rice bran/banana peel powder on weight

The weight of the samples increased noticeably as the amounts of wheat bran, rice bran, and powdered banana peel were increased. Wb7, Rb7, and Bpp7 had the highest weights, whereas control biscuits had the lowest weights. Due to the fibrous material found in the wheat bran, rice bran, and powdered banana peel, the weight of the biscuits may have risen due to the dough’s higher water demand (39).

### Color characteristics of biscuit samples

Color plays an important role in the acceptance of a food product by the consumer. Color, texture and taste are very important as far as the acceptability of biscuits is concerned. Table 5 shows the effect of all samples on L*, a*, and b* values. Highest L* value for color was observed in control 65.0 and lowest value was observed in Wb_7_, Rb_7_, and Bpp_7_ (44.3, 54.1, and 45.4). This indicates that with the increase in proportion of wheat bran, rice bran, and banana peel powder, the L* value decreases. This is because of the loss of white color of flour (37) or it may be attributed to darker color of wheat bran, rice bran and banana peel powder (44).

**TABLE 5:** Effect of wheat bran, rice bran and banana peel powder on the color value of biscuit samples.

Formulations											
	L*	a*	b*		L*	a*	b*		L*	a*	b*
Wb_2_	48.9 ± 0.005^cde^	3.9 ± 0.5^k^	27.2 ± 0.6^j^	**Rb_2_**	59.60 ± 0.04^j^	4.49 ± 0.005^ef^	20.8 ± 0.01^l^	**Bpp_2_**	63.8 ± 0.005^k^	2.4 ± 0.02^a^	17.3 ± 0.4^e^
Wb_3_	47.5 ± 0.005^bcd^	4.4 ± 0.01^l^	28.2 ± 0.005^k^	**Rb_3_**	58.81 ± 0.005^j^	4.69 ± 0.01^f^	22.7 ± 0.01^h^	**Bpp_3_**	57.4 ± 0.005^ij^	3.3 ± 0.01^b^	18.8 ± 0.005^d^
Wb_4_	46.5 ± 0.005^abc^	4.6 ± 0.01^lm^	28.5 ± 0.005^l^	**Rb_4_**	56.80 ± 0.015^hij^	5.6 ± 0.005^g^	24.6 ± 0.01^g^	**Bpp_4_**	51.01 ± 0.005^ef^	3.8 ± 0.01^c^	19.01 ± 0.005^c^
Wb_5_	45.6 ± 0.0^ab^	4.9 ± 0.5^m^	29.1 ± 0.005^l^	**Rb_5_**	55.20 ± 0.58^ghi^	5.8 ± 0.005^g^	26.2 ± 0.02^f^	**Bpp_5_**	49.08 ± 0.005^cde^	4.3 ± 0.01^d^	20.1 ± 0.01^c^
Wb_6_	44.3 ± 0.01^a^	5.3 ± 0.11^n^	29.5 ± 0.005^m^	**Rb_6_**	54.1 ± 0.0c05^gh^	7.8 ± 0.001^h^	28.3 ± 0.005^e^	**Bpp_6_**	45.4 ± 0.005^ab^	4.41 ± 0.01^d^	20.9 ± 0.01^b^
Wb_7_	43.9 ± 0.57^a^	5.5 ± 0.005^n^	30.1 ± 0.005^n^	**Rb_7_**	52.8 ± 0.005^fg^	10.2 ± 0.02^i^	30.8 ± 0.01^d^	**Bpp_7_**	45.1 ± 0.005^ab^	4.6 ± 0.01^d^	28.4 ± 0.005^a^

Control: L: 65.0 ± 0.005^def^, a: 2.3 ± 0.01^j^, b: 17.0 ± 0.005^i^. Data presented are Mean ± SD. Value with different lower case letter in column are significantly different (*p* ≤ 0.01). *Symbol of L*, a*, and b* indicates that this is the new color system.

It is evident from Table 5 that with increase in incorporation of wheat bran, rice bran, and banana peel powder a* value increased non-significantly in biscuit samples. Highest a* value was observed in Wb_7_, Rb_7_, and Bpp_7_ (15.5, 11.2, 4.6) and lowest value was observed in control 2.3. Increase in a* value within samples could be due to dark color of wheat bran, rice bran, and mostly in banana peel powder. These results are in accordance with (45). For b* value, the result indicates that maximum b* value was observed in Wb_7_, Rb_7_, and Bpp_7_ (30.1, 30.8, 28.4) while as minimum value was observed in control (17.0). This might be due to darker color of fibers caused by the addition of banana peel powder is due to phytochemical compounds owned banana peels. These results are in close findings with Ajila et al. (7) and Hernawati et al. (46). The higher value of a* for biscuit samples could be due to the Maillard reaction which is taking place between protein of milk precipitates and reducing sugar. Also, the decrease in L* and increase in a* value are also indicator of browning progress (47). The b* value indicated the yellowness of the biscuit. Maillard browning and caramelization of sugar are considered to produce brown pigments during baking (48).

### Effect on textural quality of biscuit samples

Texture of food relates to the mechanical work that occurs in food processing operations, as they do when breakdown of food in the consumer’s mouth. For example, it is preferable to weaken the structure during mastication so that it will appropriately dissolve when pressures are applied. Compression test type or Texture Profile Analysis (TPA) is the required test for hardness. A texture analyzer was used to evaluate hardness. Table 6 lists the average values for the hardness of biscuit samples.

**TABLE 6:** Effect of wheat bran, rice bran and banana peel powder on hardness (N) value of biscuit samples.

Formulations					
Wb_2_	9.17 ± 0.01^hi^	**Rb_2_**	8.3 ± 0.01^de^	**Bpp_2_**	5.3 ± 0.01^a^
Wb_3_	9.22 ± 0.01^i^	**Rb_3_**	8.9 ± 0.01^f^	**Bpp_3_**	5.9 ± 0.01^b^
Wb_4_	9.55 ± 0.08^k^	**Rb_4_**	9.5 ± 0.04^k^	**Bpp_4_**	6.5 ± 0.04^c^
Wb_5_	9.90 ± 0.42^l^	**Rb_5_**	10.3 ± 0.01^m^	**Bpp_5_**	8.3 ± 0.02^d^
Wb_6_	10.3 ± 0.10^m^	**Rb_6_**	10.5 ± 0.01^n^	**Bpp_6_**	8.5 ± 0.02^e^
Wb_7_	10.5 ± 0.04^n^	**Rb_7_**	11.5 ± 0.01^l^	**Bpp_7_**	9.5 ± 0.01^g^

Control: 5.2 ± 0.11^a^. Data presented are Mean ± SD. Value with different lower case letter in column are significantly different (*p* ≤ 0.01).

### Hardness

Effect of fiber incorporation on breaking strength (N) values of biscuit samples are showed in Table 6. The breaking strength, which is the force required to break the biscuits increased significantly (*p* ≤ 0.01) from control to Wb_7_, Rb_7_, and Bpp_7_ indicating an increase in the hardness of biscuits with the addition of fibers. The lowest value of breaking strength was found in control (5.2) and the highest value was found in Wb_7_ (10.5), Rb_7_ (11.5), and Bpp_7_ (9.5). The increase in breaking strength of biscuits may be attributed to presence of soluble dietary fiber that has higher water holding capacity which results in increased dough viscosity leading to the increase in breaking strength of biscuits.

The hardness of biscuits was as a result of development of gluten network. Gluten promotes the network development by attracting the water molecules. Kumari and Grewal (49) reported similar results for hardness due to incorporation of carrot pomace. Ajila et al. (7) noticed that biscuits prepared from flour containing 20 per cent mango peel powder (MPP) had a breaking strength of 1.97 kg as compared to 0.88 kg of control biscuits. Erinc et al. (50) also reported that addition of wheat bran resulted in harder texture of the biscuits. They further reported that beyond 30 percent level of bran addition, the biscuit became slightly harder and darker in color.

### Optical microscopy of optimized biscuit samples

The impact of the ingredients on the sample structure may be seen using optical microscopy. The samples prepared with banana peel powder, wheat bran, and rice bran displayed an irregular structure at the levels of magnification used in this study, as shown in Figure 3, which could be explained by the replacement of gluten protein by the banana peel powder and bran (rice and wheat). The control contained a compact and uniform structure. During baking of dough, the lower gluten quantity prevents the creation of the ideal networks (51). The reduction in biscuit sample diameter and thickness may be connected to the decline in gluten network development. The height and volume of the muffins were similarly decreased when some of the flour was substituted with other ingredients, according to Lee et al. (52). Leiva-Valenzuela et al. (27) used optical microscopy to examine samples of biscuits in which dietary fiber had been used in place of some of the flour. The micrographs, particularly with high amounts of fiber, showed an uneven structure and bubbles started to form toward the end of baking, supporting our findings. Microscopic observations of the biscuits enriched with wheat bran/rice bran and banana peel powder also showed an intermediate degree of starch gelatinization (53).

Among the different biscuit samples, the highest moisture was observed in control (4.8) and followed by other samples. The moisture content of fiber incorporated biscuits showed a significant (*p* ≤ 0.01) difference within treatments on different days. The moisture content of approved formulations increased non-significantly (*p* ≤ 0.01) from first day of storage to 60th day, going from 3.40 to 3.91% in Wb4, 3.93 to 4.52% in Rb4, and 3.13 to 3.66% in Bpp3. This rise in moisture content may be caused by ambient moisture, which continues to affect the biscuit samples even after packaging, or by the hygroscopic properties of refined wheat flour, rice bran, wheat bran, and banana peel powder, which absorbed atmospheric moisture during storage.

The increased moisture absorption of biscuits containing bran during storage may be related to hygroscopic nature of wheat bran (54).

In the biscuit samples, the ash content showed a non-significant decreasing trend (*p* ≤ 0.01). The ash content in the optimized biscuit samples dropped from Control (0.48–0.29%), Wb4 (0.86–0.67%), Rb4 (0.95–0.75%), and Bpp3 (1.15–0.92%) at the end of 60th day of storage. The decrease in ash content of biscuit samples over time may be related to moisture absorption, which reduces the concentration of other components. These conclusions are supported by the finding of Chakraborty et al. (55). Mineral losses can be caused by reducing sugars reacting chemically with proteins or amino acids under the influence of heat to produce compounds that bind minerals. These browning reaction products can retain their mineral-binding qualities because they are more resistant to digestion. Water also contains significant volumes of dissolved minerals. Due to the hygroscopic nature of the substance, this also causes mineral loss throughout the course of the storage term. Similar to the changes in food composition as a result of packaging, mineral bio-availability can also change.

In samples of the improved biscuits, a substantial (*p* ≤ 0.01) drop in fat content was observed during storage. These biscuit samples’ average fat content during the course of storage was observed to be in the range of 15.89–15.69% for Control, 16.32–16.12% for Wb4, 16.83–16.52% for Rb4, and 16.38–16.17% for Bpp3. The lipase enzyme’s activity, which breaks down fat into free fatty acids and glycerol in the presence of catalysts like moisture, light, and heat, or the hydrolysis of triglycerides, may be to reason for the reduced fat content during storage. These results are consistent with those of Pratyush et al. (56), who found that cookies enriched with varying amounts of pumpkin powder had a lower fat content after being stored.

In samples of optimized biscuits, the protein content exhibited a non-significant tendency of declining over storage. It was discovered that the protein content dropped from 6.35 to 6.14% in Control, 7.24 to 7.03% in Wb4, 7.95 to 7.53% in Rb4, and 6.77 to 6.32% in Bpp3. This drop in protein concentration may be caused by the protease enzyme’s hydrolysis of peptide bonds, which splits protein molecules during storage. Similar decrease of protein content with storage period was reported by Nadarajah and Thevaki (57) in protein enriched biscuits.

During the storage period, the fiber content of the biscuit samples considerably dropped. Table 7 displays the changes in biscuits’ fiber content with storage. Sujirtha and Thevaki (58) observed a similar decline in fiber content with storage time in their study on the effects of storage on the qualitative features of coconut flour-based biscuits.

**TABLE 7:** The changes in moisture content of biscuits during storage period.

Optimized samples	Time	Moisture	Ash	Fat	Protein	Fiber
Control	**0th day**	4.8 ± 0.01^efA^	0.48 ± 0.02^bC^	15.89 ± 0.02^aC^	6.35 ± 0.01^aC^	0.45 ± 0.01^aC^
	**30th day**	4.93 ± 0.0.01^cB^	0.37 ± 0.02^aB^	15.80 ± 0.01^bB^	6.25 ± 0.01^aB^	0.38 ± 0.01^aB^
	**60th day**	4.95 ± 0.01^cC^	0.29 ± 0.02^aA^	15.69 ± 0.02^bA^	6.14 ± 0.01^aA^	0.33 ± 0.02^aA^
Wb_4_	**0th day**	3.40 ± 0.005^cde^	0.86 ± 0.03^eC^	16.32 ± 0.02^dC^	7.24 ± 0.01^fC^	1.25 ± 0.02^gC^
	**30th day**	3.53 ± 0.01^bB^	0.75 ± 0.01^aB^	16.23 ± 0.01^aB^	7.14 ± 0.01^bB^	1.18 ± 0.005^aB^
	**60th day**	3.91 ± 0.01^Bc^	0.67 ± 0.01^aA^	16.12 ± 0.01^aA^	7.03 ± 0.01^bA^	1.13 ± 0.02^aA^
Rb_4_	**0th day**	3.93 ± 0.15^gA^	0.95 ± 0.03^fC^	16.83 ± 0.02^iC^	7.95 ± 0.03^lC^	1.29 ± 0.01^hC^
	**30th day**	4.21 ± 0.01^cA^	0.85 ± 0.02^bB^	16.68 ± 0.05^cB^	7.73 ± 0.01^cB^	1.23 ± 0.01^bB^
	**60th day**	4.52 ± 0.01^cA^	0.75 ± 0.005^bA^	16.52 ± 0.01^cA^	7.53 ± 0.01^cA^	1.18 ± 0.02^aA^
Bpp_3_	**0th day**	3.13 ± 0.02^bcA^	1.15 ± 0.02^gC^	16.38 ± 0.02^dC^	6.77 ± 0.01^cC^	1.55 ± 0.005^jC^
	**30th day**	3.36 ± 0.02^aB^	1.04 ± 0.02^cB^	16.240.01^aB^	6.53 ± 0.01^aB^	1.42 ± 0.01^cB^
	**60th day**	3.66 ± 0.01^aC^	0.92 ± 0.02^cA^	16.17 ± 0.01^bA^	6.32 ± 0.01^aA^	1.32 ± 0.02^bA^

Data presented are mean ± SD. Values with same upper case in rows are significantly different (*p* ≤ 0.01). Values with different lower case in column are significantly different (*p* ≤ 0.01).

## Conclusion

Biscuits are one of the popular cereal food types, consumed as breakfast items and have a pleasant taste, are ready to eat, accessible cost, easily available and have a long shelf life. The result showed that fiber and protein content increases as more Wb, Rb, and Bpp were incorporated. The sensorial attributes of biscuits with Wb (15%), Rb (15%), and Bpp (10%) substitution level were more preferred. Thus, supplementation of Wb, Rb, and Bpp in baked products formulation is suitable for baking process and enrichment. They are possibly being utilized both as functional ingredients and as partial ingredients in bakery products because of their ability to improve nutritional quality and health issues without compromising the palatability. The production of biscuits enriched with wheat bran, rice bran, and banana peel powder can be considered as an alternative way to include this health promoter fiber in human nutrition.

## Data availability statement

The original contributions presented in this study are included in the article/supplementary material, further inquiries can be directed to the corresponding author/s.

## Author contributions

WSA: study conception and design. AHD: supervision and data analysis. IZ: data collection and draft manuscript preparation. SF and NA: interpretation of results. AHD and RP: conceptualization. WSA, IZ, NA, and SF: methodology. WSA, IZ, NA, SF, and GJ: investigation. RP, AVR, JMR, and GJ: software. RP, AVR, JMR, and MT: resources. WSA, IZ, NA, SF, GJ, and RP: writing—original draft preparation. RP, AVR, JMR, and MT: writing—review and editing. AHD, RP, AVR, JMR, and MT: supervision. AVR, JMR, and MT: funding acquisition. All authors reviewed the results and manuscript preparation.
